# Vicinal abasic site impaired processing of a Tg:G mismatch and 8-oxoguanine lesions in three-component bistranded clustered DNA damage†

**DOI:** 10.1039/c8ra01992d

**Published:** 2018-05-16

**Authors:** Bhavini Kumari, Pravin Jha, Kislay K. Sinha, Prolay Das

**Affiliations:** Department of Chemistry, IIT Patna Bihta Patna-801103 Bihar India prolay.das@gmail.com; Department of Biotechnology, NIPER Hajipur Bihar India

## Abstract

The occurrence of 7,8-dihydro-8-oxo-2′deoxyguanosine (8-oxodG), thymine glycol:guanine (Tg:G) mismatch and abasic site DNA damage lesions in close proximity induce repair refractive multicomponent clustered DNA damage. Herein, the influence of abasic sites in the processing of 8-oxodG lesion and Tg:G mismatch bistranded cluster is evaluated. Abasic sites are found to impart conformational destabilization that appreciably hinders the repair activity of the other lesions whenever present in a cluster combination. The repair process reduces the formation of double strand breaks (DSBs) and renders this three-lesion combination a non-DSB forming cluster. The stability of the DNA duplex harbouring these three lesions is highly compromised due to altered base helicity and base stacking phenomena leading to impaired repair.

## Abstract 2

The occurrence of 7,8-dihydro-8-oxo-2′deoxyguanosine (8-oxodG), thymine glycol:guanine (Tg:G) mismatch and abasic site DNA damage lesions in close proximity induce repair refractive non-DSB cluster.

## Graphical abstract

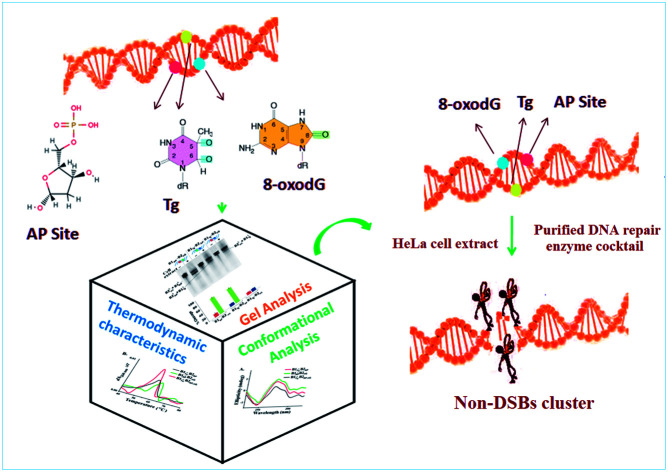

Damaged lesions in deoxyribonucleic acid (DNA) are ubiquitous due to the exposure of cells to various exogenous sources, including ionizing radiation and toxins, as well as endogenous metabolism.^1,2^ 5-Methyl cytosine (5-mC) residues in DNA are vulnerable to radiation-induced oxidation that could generate a Tg:G mismatch.^3^ In addition, stripping of purine or pyrimidine bases giving rise to potentially mutagenic abasic sites is a familiar event.^4^ 8-oxodG is one of the most common DNA damage lesions that plays a critical role in mutagenesis and carcinogenesis.^5^ However, the present understanding of the repair pattern of three-component multilesion clustered DNA damage is very limited, particularly in clusters containing a Tg:G mismatch and 8-oxodG in the vicinity of abasic sites. Herein, for the first time, we demonstrate the role of abasic sites in the repair of a Tg:G mismatch and 8-oxodG present in a three component bistranded cluster environment.

The DNA repair machinery in our cells has the huge responsibility of repairing chemically unrelated lesions in order to ensure the smooth functioning of cellular processes and maintain genomic integrity.^6^ Evidently, the challenge for the DNA repair machinery is even greater when multiple lesions are present in close proximity to one another giving rise to multicomponent clustered DNA damage, often reported as a major outcome of exposure to ionizing radiation.^7–12^ Subsequent repair takes place through the intervention of various well recruited enzymes of the base excision repair (BER) pathway.^13^ Specifically, in human cells, the repair of abasic sites, 8-oxodG and Tg lesions involves apurinic/apyrimidic endonuclease (APE1), oxoguanine glycosylase (hOGG1) and hNTH1 enzymes, respectively, in the initial stages following the BER pathway.^14,15^ The attempted repair of bistranded clusters often leads to the generation of potentially lethal double strand breaks (DSBs) that could lead to deletions, translocations, and fusions.^16^ Reportedly, bistranded clustered abasic sites are processed to yield DSBs in *E. coli* leading to cytotoxicity.^17^ In contrast, most clustered damage systems adopt a hierarchal repair strategy to lessen the DSB formation while attempting repair.^18^ The close proximity of the lesions and their structural and chemical differences decides the sequence in which the lesions are processed in the hierarchy. This competition is based on the kinetics of damage recognition and the removal of damaged bases.^19^ Hence, radiation induced multicomponent clustered DNA damage in cells can be mainly categorized as DSB clustered and non DSB clustered lesions. These are purely lesions and their repair path dependent phenomena have been witnessed in both *in vitro* and *in vivo* studies.^20^ Efforts have been made to uncover the preferred pathway for the repair of these oxidised lesions in tandem or in the presence of a neighbouring oxidised lesion by lesion specific enzymes or cell extracts.^21,22^

A Tg:G mismatch and 8-oxodG lesion in a bistranded cluster environment have been repaired quite efficiently in the presence of one another in a oligonucleotide and nucleosome model.^23^ Herein, the influence of the vicinal presence of an abasic site near an 8-oxodG lesion and Tg:G mismatch was evaluated by estimating the cleavage efficiency of the repair specific enzymes hOGG1 and hNTH1, respectively, in a two lesion, as well as a three lesion, oligonucleotide model. Our studies show that the abasic site presents a significant hurdle in the efficient repair of the Tg:G mismatch and 8-oxodG lesion by introducing conformational destabilization in the DNA that prevents proper interaction of the lesion specific enzymes with the lesions.

## Results and discussion

B1_AP_-B2_oG_ and B1_Tg_-B2_AP_ (Table 1) represent two component cluster-containing oligomer DNA duplexes that have Tg:G mismatch and 8-oxodG lesions. In B1_Tg_-B2_AP,oG_, the Tg:G mismatch is present at the −4 position from the 8-oxodG lesion and the +3 position from the abasic site. All information regarding material, procedure and instrumentation used in present study has been provided in ESI.† The cleavage efficiency was evaluated in the oligomers and other systems with an optimized amount of lesion specific purified enzymes and cell extracts (Fig. S1 and S2†) and compared with oligomers that have one Tg:G mismatch and an 8-oxodG lesion that showed *ca.* 74% and 78% cleavage with hNTH1 and hOGG1, respectively (Fig. S3†).

**Table 1:** DNA duplexes used in the present study^a^

B1-B2_AP_	5′PO_4_^3−^ AAC TCG GTG TTA AAG CCT GCA ACA CTC CGT GCG GAG AAT ACT CTC GGT GTT
	3′OH-GC CAC AAT UTC GGA CGT TGT GAG GCA CGC CTC TTA TGA GAG CCA CAA TCTC
B1_AP_-B2_oG_	5′PO_4_^3−^ AAC TCG GTG TTA AAG UCT GCA ACA CTC CGT GCG GAG AAT ACT CTC GGT GTT
	3′OH-GC CAC AAT TTC GGA C_O_GT TGT GAG GCA CGC CTC TTA TGA GAG CCA CAA TCTC
B1_Tg_-B2_AP_	5′PO_4_^3−^ AAC TCG GTG TTA AAG T_g_CT GCA ACA CTC CGT GCG GAG AAT ACT CTC GGT GTT
	3′OH-GC CAC AAT UTC GGA CGT TGT GAG GCA CGC CTC TTA TGA GAG CCA CAA TCTC
B1_Tg_-B2_AP,oG_	5′PO_4_^3−^ AAC TCG GTG TTA AAG T_g_CT GCA ACA CTC CGT GCG GAG AAT ACT CTC GGT GTT
	3′OH-GC CAC AAT UTC GGA C_O_GT TGT GAG GCA CGC CTC TTA TGA GAG CCA CAA TCTC
B1_Tg_-B2_THF_	5′PO_4_^3−^ AAC TCG GTG TTA AAG TgCT GCA ACA CTC CGT GCG GAG AAT ACT CTC GGT GTT
	3′OH-GC CAC AAT XTC GGA CGT TGT GAG GCA CGC CTC TTA TGA GAG CCA CAA TCTC
B1_THF_-B2_oG_	5′PO_4_^3−^ AAC TCG GTG TTA AAG XCT GCA ACA CTC CGT GCG GAG AAT ACT CTC GGT GTT
	3′OH-GC CAC AAT TTC GGA C_O_GT TGT GAG GCA CGC CTC TTA TGA GAG CCA CAA TCTC
B1_Tg_-B2_THF,oG_	5′PO_4_^3−^ AAC TCG GTG TTA AAG T_g_CT GCA ACA CTC CGT GCG GAG AAT ACT CTC GGT GTT
	3′OH-GC CAC AAT XTC GGA C_O_GT TGT GAG GCA CGC CTC TTA TGA GAG CCA CAA TCTC
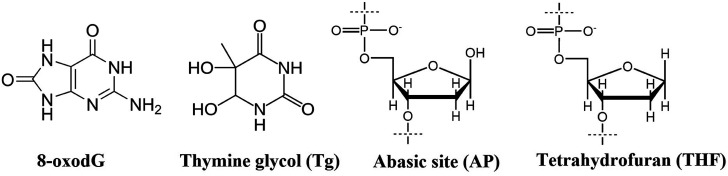	

a Tg: thymine glycol, AP: abasic site, oG: 8-oxodG, U: uracil, THF: tetrahydrofuran (abasic site analog), denoted by an X. Uracils serve as the site for abasic site formation following UDG treatment.

The processing of a solitary 8-oxodG lesion using a cocktail of hOGG1 and APE1 enzymes and a solitary Tg:G mismatch using a cocktail of hNTH1 and APE1 enzymes was also evaluated (Fig. S4†). The generation of a bound complex was observed in the case of the 8-oxodG lesion. This bound complex is the outcome of the complexation between hOGG1 and a pre-formed abasic site due to the glycosylase action of hOGG1. A relative decrease in the amount of the bound complex with a proportionate increase in the amount of APE1 in the enzyme cocktail was observed. This suggests that the rate limiting step here is the dissociation of hOGG1 from the abasic site produced from the 8-oxodG lesion due to its glycosylase activity. APE1 enhances the turnover of hOGG1 through competition for the same abasic site produced as an intermediate and further enhances the release of the hOGG1 enzyme from the 8-oxodG lesion.^24,25^ No such bound complex formation and proportionate decrease in its intensity has been observed in the case of the Tg:G mismatch with a cocktail of hNTH1 and APE1 enzymes.

**Fig. 1:**
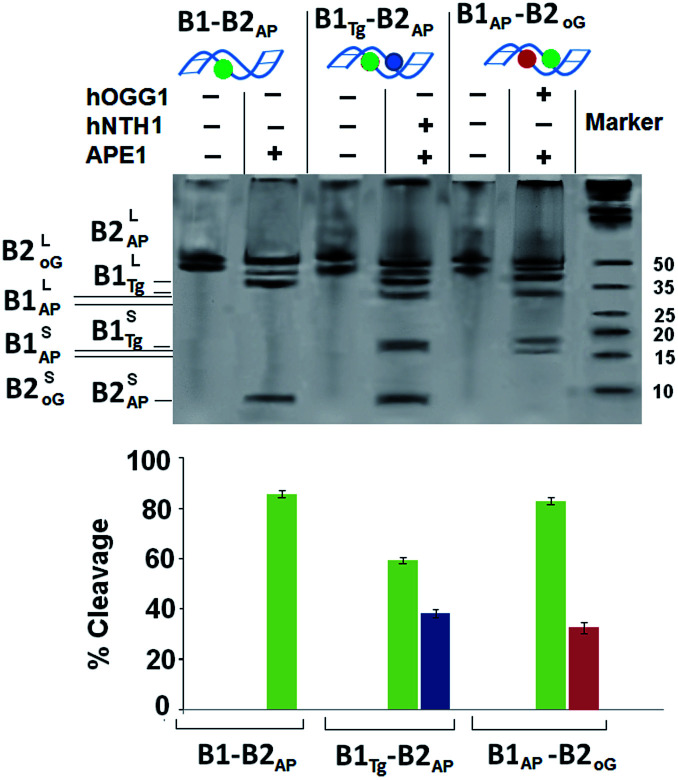
The 20% denaturing PAGE (polyacrylamide gel electrophoresis) showing SSB formation in B1-B2_AP_ with APE1, in B1_AP_-B2_oG_ with a cocktail of APE1 and hOGG1, and in B1_Tg_-B2_AP_ with hNTH1 and APE1 with their respective control. Three independent measurements (*n* = 3) were used to calculate the standard deviation (SD). The values of the error bars represent the mean ± SD. The gel ran at 200 V for 2 h and was stained with sybr gold®.

For B1_AP_-B2_oG_, an enzyme cocktail of hOGG1 and APE1 was used that showed a drastic reduction in the cleavage of the 8-oxodG lesion in the presence of the abasic site from 78% to 32%. However, incision efficiency of the abasic site was maintained at ∼82%, which indicates the unhindered interaction of APE1 with the abasic site. The amount of cleavage resulting from the Tg:G mismatch and abasic site in B1_Tg_-B2_AP_, by the action of a cocktail of hNTH1 and APE1 enzymes showed ∼38% and ∼58% cleavage, respectively, for the Tg and abasic site lesions (Fig. 1). Thus, the presence of abasic site lesions slows the processing of the Tg:G mismatch considerably, even when the three bases are present in opposing strands. Moreover, the efficiency of the action of APE1 on the abasic site was also found to be compromised due to the vicinal presence of the Tg:G mismatch. Thus, it is clear that the processing of the Tg:G mismatch and the abasic site is mutually distracting.

In the present study, a cocktail of hOGG1 and APE1 enzymes shows only a 32% cleavage of the 8-oxodG lesion in a B1_AP_-B2_oG_ DNA duplex. To explore the potential cause behind the restricted cleavage of the 8-oxodG lesion, a band shift assay was performed. The band shift assay shows the unhindered approach of hOGG1 towards the solitary 8-oxodG lesion in B1-B2_oG_. B1_Tg_-B2_oG_ showed an intermediate behaviour in the native PAGE, where some hindrance in the interaction between hOGG1 and its substrate was evident (Fig. S5†). However, restricted binding of the hOGG1 enzyme was observed in the oligomer B1_AP_-B2_oG_. This decreased intensity of the bound complex points towards the disrupted helicity enforced due to the presence of the abasic site resulting in significant hindrance of the approach of hOGG1 towards the 8-oxodG lesion in the two-component cluster embracing an abasic site.^26^ The processing of B1_AP_-B2_oG_ with a cocktail of hOGG1 and APE1 enzymes leads to less competition for the abasic site due to the glycosylase action of the hOGG1 enzyme and the concurrent occurrence of another abasic site in the vicinity. Additionally, the hindered approach of the hOGG1 enzyme towards the 8-oxodG lesion leads to the compromised formation of a bound complex. Turnover of the hOGG1 enzyme by the APE1 enzyme solely depends on the concentration of the bound complex as the APE1 enzyme releases the hOGG1 enzyme from the 8-oxodG lesion site. Both of the phenomena discussed above were responsible for the restricted cleavage by the hOGG1 enzyme.

Approximately 82% and 58% cleavage of the abasic site was evident in the B1_AP_-B2_oG_ and B1_Tg_-B2_AP_ DNA duplexes with enzyme cocktails of the respective repair specific enzymes. Both the hOGG1 and hNTH1 enzymes are bifunctional glycosylases that possess major glycosylase, as well as mild AP lyase, activity under physiological conditions. Therefore, these results might have the compounded outcome of abasic site cleavage due to the AP lyase action of these bifunctional glycosylases and the APE1 enzyme. To determine the actual cleavage of the abasic site due to the APE1 enzyme, the abasic site analog tetrahydrofuran (THF) was used instead of the abasic site in a DNA duplex in a control experiment. This chemically stable synthetic analog is a substrate for the APE1 enzyme and is very efficiently cleaved by it and at the same time remains totally unaffected by the AP-lyase action of the hOGG1 and hNTH1 enzymes. An approximate 6% decrease in the cleavage of THF was evident in the case of B1_THF_-B2_oG_, whereas no significant decrease in the cleavage was observed for the B1_Tg_-B2_THF_ DNA duplex, with respect to their abasic site counterparts (Fig. S6†).

**Fig. 2:**
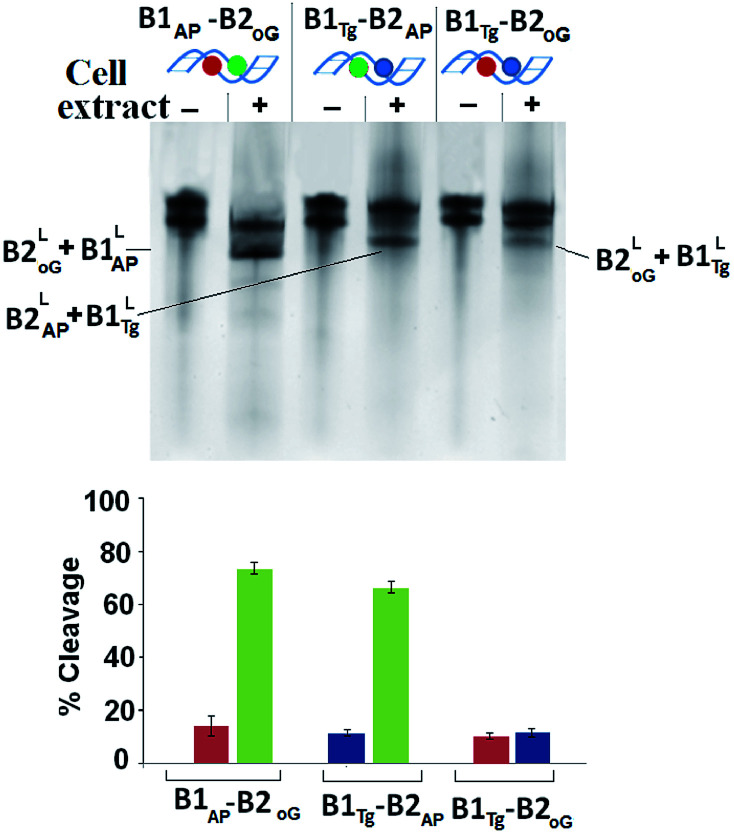
The 20% denaturing PAGE showing SSB formation in B1-B2_AP_, B1_AP_-B2_oG_ and B1_Tg_-B2_AP_ due to optimized amounts of cell extracts with their respective controls. Three independent measurements (*n* = 3) were used to calculate the standard deviation (SD). The values of the error bars represent the mean ± SD. The gel ran at 200 V for 2 h and was stained with sybr gold®.

HeLa cells were obtained from National Center for Cell Sciences, Pune (India). The information regarding processing and optimization of cell extract has been provided in ESI.† The repair pattern of two-lesion clustered DNA (B1_Tg_-B2_AP_ and B1_AP_-B2_oG_) was also studied using a HeLa cell extract containing the repair enzymes in relevant concentrations. A solitary abasic lesion in B1-B2_AP_ was efficiently cleaved (88%) with the cell extract. However, the cleavage efficiency values of the 8-oxodG lesion and the abasic site in B1_AP_-B2_oG_ was found to be only ∼10% and ∼75%, respectively. In the B1_Tg_-B2_AP_ strand, incision efficiency values of ∼12% and ∼70% were obtained for the Tg mismatch and the abasic site, respectively (Fig. 2). Thus, for B1_Tg_-B2_AP_ and B1_AP_-B2_oG_, the cleavage pattern with the HeLa cell extract was similar to those of the purified enzyme cocktails composed of the hNTH1-APE1 and hOGG1-APE1 enzymes respectively, with significantly reduced efficiency. The excision efficiency of a particular damaged nucleobase may to some extent depend on the amount of the repair specific DNA *N*-glycosylases/lyases present in the cell extract. Thus, this contrasting repair trend in the Tg:G mismatch and 8-oxodG lesion may in part be attributed to the difference in the proportion of the repair enzymes present in the purified enzyme cocktail and HeLa cell extract. The inefficient processing of Tg may also be due to the reduced glycosylase activity of hNTH1, which is approximately seven times less when G serves as an opposing base to Tg rather than A.^27^

**Fig. 3:**
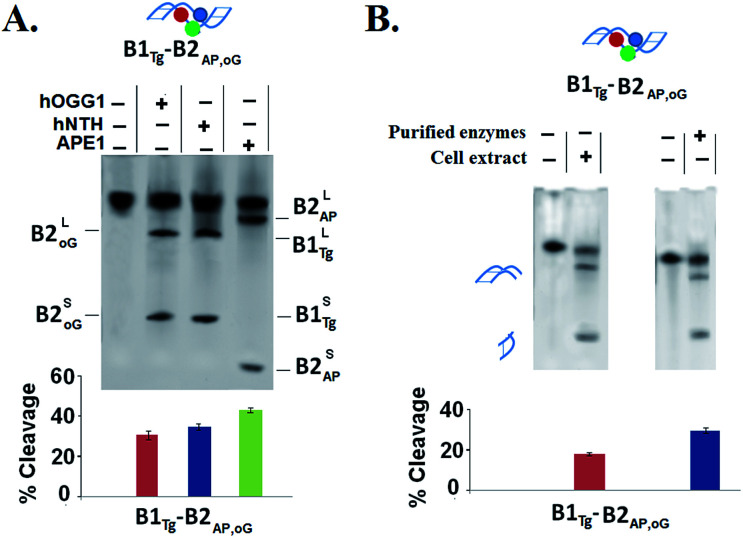
(A) The 20% denaturing PAGE showing SSB formation due to the cleavage of specific single lesions in B1_Tg_-B2_AP,oG_ with the hOGG1, hNTH1 and APE1 purified enzymes at 200 V, (B) the 20% native PAGE shows DSBs in B1_Tg_-B2_AP,oG_ with the purified enzyme cocktail of hOGG1, hNTH1 and APE1, and the HeLa cell extract at 200 V. The larger and smaller fragments represent the upper and lower bands due to the simultaneous incision of lesions owing to their bistranded location. Three independent measurements were used to calculate the standard deviation (SD). The values of the error bars represent the mean ± SD. Both gels ran at 200 V for 2 h and were stained with sybr gold®.

The oligomers with the complex three-lesion cluster B1_Tg_-B2_AP,oG_ were processed separately with single lesion specific enzymes *i.e.* hOGG1, hNTH1 and APE1. Herein, a ∼30% cleavage of 8-oxodG by hOGG1, 34% of Tg by hNTH1 and 42% cleavage of abasic sites by APE1 were observed. Evidently, the cleavage efficiency of the individual enzymes was found to be heavily compromised due to the introduction of abasic sites in this cluster (Fig. 3A). A purified enzyme cocktail composed of hOGG1, hNTH1 and APE1 enzymes also showed the compromised generation of DSBs in B1_Tg_-B2_AP,oG_ (Fig. 3B). DSBs were also analyzed in similar DNA duplexes which have THF (B1_Tg_-B2_THF,oG_) instead of abasic sites to evaluate the AP-lyase action of the hOGG1 and hNTH1 enzymes if any, where no significant changes in the DSB formation were observed.

A further decline in the processing of the three lesion cluster in B1_Tg_-B2_AP,oG_ by the HeLa cell extract was observed compared to that of the purified enzyme cocktail. B1_Tg_-B2_AP,oG_ showed a lower amount of DSB (∼18%) formation with the cell extract compared to the DSBs obtained with the purified enzyme cocktail (∼30%). This reveals an important difference regarding the processing of the multicomponent clusters by the purified enzyme and cell extract. Our results point to the fact that the present multiclustered damage in B1_Tg_-B2_AP,oG_ is a non DSB forming cluster. In this present cluster, efficient spatial and sequential coordination is achieved among the repair enzyme for circumventing DSBs while attempting repair of this non DSB clustered damage. The overall effect is the persistence of long-lived repair intermediates for an appreciable amount of time.

The cell extract was complimented with a repair buffer containing dNTPs (deoxyribonucleotide triphosphate) to monitor the repair of abasic sites over a 30 min time frame. The B1_AP_-B2_oG_ and B1_Tg_-B2_AP_ strands showed ∼35% and 20% restoration of the oligonucleotides over 30 min indicating efficient repair (Fig. S7 and S8†). However, such type of repair and relevant restoration of the cleaved lesions were not observed for B1_Tg_-B2_AP,oG_ (Fig. S9†). This shows that for complex multicomponent clustered damages, DNA repair activity is being carried out in a restricted fashion. Reduced generation of DSBs in B1_AP_-B2_oG_, B1_Tg_-B2_AP_ and B1_Tg_-B2_AP,oG_ points towards the sequential repair of the lesions in these oligomers.

**Fig. 4:**
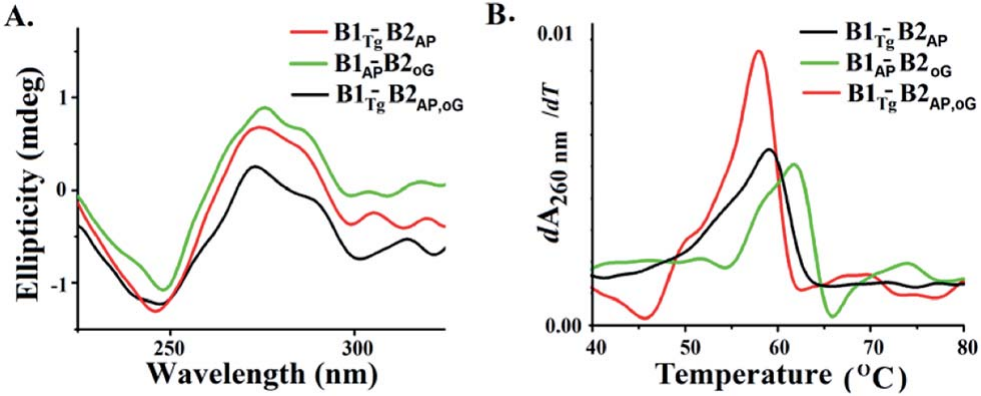
(A) CD spectra of B1_Tg_-B2_AP_, B1_AP_-B2_oG_ and B1_Tg_-B2_oG,AP_ (B) first differential of the thermal melting curve for B1_Tg_-B2_AP_, B1_AP_-B2_oG_ and B1_Tg_-B2_oG,AP_.

B-DNA possesses characteristic positive peaks at 275 nm and a negative peak at 245 nm in the circular dichroism (CD) spectra corresponding to its helicity and base stacking, respectively.^28^ Noteworthy changes in the amplitude of the positive and negative peaks were observed in the CD spectra of B1_AP_-B2_oG_, B1_Tg_-B2_AP_ and B1_Tg_-B2_AP,oG_ (Fig. 4A). This is indicative of the altered π–π stacking and base pairing due to the presence of clusters of damage lesions.^29^ The maximum decrease in the amplitude of the peaks in the CD spectra was observed for B1_Tg_-B2_AP,oG_ followed by B1_Tg_-B2_AP_ and B1_AP_-B2_oG_ (Fig. S10†).^30,31^ In B1_Tg_-B2_AP_, the decrease in the amplitude of the peak can be attributed to the extrahelical nature of Tg.^32^ The presence of Tg, 8-oxodG and abasic sites in B1_Tg_-B2_AP,oG_ cause a significant decrease in the amplitude of the positive peaks, compared to those of B1_AP_-B2_oG_ and B1_Tg_-B2_AP_. This accounts for the more prominent disturbed base stacking due to the presence of the three types of lesions. Considerable helix destabilization was observed due to the introduction of abasic sites in the vicinity of the other lesions.

Thermodynamic characterization of the melting transitions in a DNA duplex provides information regarding the stability and temperature-dependent melting behavior (*T*_m_).^33,34^ The *T*_m_ value of B1-B2_AP_ was found to be 63.9 °C and served as the control (Fig. S11†). The *T*_m_ value of B1_Tg_-B2_AP_ was found to be ∼59 °C and a value for B1_oG_-B2_AP_ of 61.8 °C was recorded (Fig. 4B and S12†). The lowest *T*_m_ value was observed for the three lesion cluster containing B1_Tg_-B2_AP,oG_ at 57.8 °C. Free energy (Δ*G*) differences of 100 cal mol^−1^, 300 cal mol^−1^ and 900 cal mol^−1^ were calculated from the van't Hoff plots for B1_AP_-B2_oG_, B1_Tg_-B2_AP_ and B1_Tg_-B2_AP,oG_, respectively, using B1-B2_AP_ as a control (Table 2 and Fig. S13 to S16†). The presence of three lesions results in maximum thermal and thermodynamic destabilization due to the altered base stacking and decreased stability. The free energy difference of 1.7 kcal mol^−1^ between the B1_Tg_-B2_AP,oG_ and B1_Tg_-B2_oG_ duplexes is accountable for the equilibrium preference of 15.8 : 1 between them.

**Table 2:** Thermodynamic parameters for the formation of the DNA duplexes

Sequence	Melting temperature *T*_m_^a^^,^^b^ (°C)	Δ*G*^0^_37_^b^ (kcal mol^−1^)	Δ*H*^0^^b^ (kcal mol^−1^)	Δ*S*^0^^b^ (cal mol^−1^ K^−1^)
B1-B2_AP_	63.9 ± 0.6	−17.1 ± 1.3	−148.4 ± 04.3	−423.8 ± 09.7
B1_AP_-B2_oG_	61.8 ± 0.2	−17.0 ± 1.9	−144.4 ± 05.3	−411.1 ± 11.1
B1_Tg_-B2_AP_	58.9 ± 0.3	−16.8 ± 1.5	−142.8 ± 04.8	−406.5 ± 10.8
B1_Tg_-B2_AP,oG_	57.8 ± 0.5	−16.2 ± 1.7	−133.1 ± 05.1	−376.9 ± 11.2

a The DNA duplex concentration was 5.0 μM.

b Three independent measurements were used to calculate the standard deviation.

## Conclusions

Tg:G mismatches in a three component cluster containing abasic and 8-oxoG lesions have not been studied before. Herein, we tried to evaluate the difference in the processing efficiency of a purified enzyme cocktail and cellular extract in 2–3 component bistranded clustered DNA damage. In the case of B1_Tg_-B2_AP_ and B1_AP_-B2_oG_, independent processing was not observed with hNTH1-APE1 and hOGG1-APE1 cocktails, respectively. Cleavage of Tg in B1_Tg_-B2_AP_, as well as 8-oxodG in B1_AP_-B2_oG_, is compromised due to the presence of an abasic site in the opposing strand. Both Tg and 8-oxodG lesions are converted to abasic sites by the action of hNTH1 and hOGG1. The presence of APE1 in the enzyme cocktail ensures the cleavage of the already present abasic sites as well as those generated *in situ* from Tg and 8-oxodG using the hNTH1 and hOGG1 enzymes, respectively. Helix destabilization, as evident from the CD spectra and thermal melting studies of B1_AP_-B2_oG_ and B1_Tg_-B2_AP_ strands, prevent APE1 from acting properly on its substrate. Reportedly, in a bistranded cluster, the processing of a second abasic site is greatly reduced by the conformational destabilization introduced by the cleavage of the first abasic site.^35,36^ In both B1_Tg_-B2_AP_ and B1_AP_-B2_oG_, irrespective of the order of cleavage of the Tg or 8-oxodG lesions and the abasic sites, conformational destabilization is introduced due to the cleavage of one of the abasic sites that interrupts the processing of the other lesion. The overall result is a drastic reduction in the cleavage efficiency by the enzyme cocktail. This restricted cleavage ensures the formation of fewer DSBs. While efficient restoration was observed for two component clusters, such a phenomenon was absent for the three-component cluster. From this observation, it can be conferred that the vicinal presence of three lesions greatly hinders the repair process and renders the three-component cluster a non-DSB forming one. In this non-DSB cluster, efficient spatial and sequential coordination was achieved among the repair enzymes for circumventing DSBs while attempting repair. The notion of repair refractivity of multicomponent clustered DNA damage arises from conformational destabilization in the DNA at the clustered site that prevents the enzymes concerned from working properly on the specific substrates. In the current combination of clustered lesions, the presence of abasic sites dictates this process through conformational destabilization that prevents the enzymes from processing their respective substrates with full efficiency. The design and sequence of the oligomer used here has restriction endonuclease compatible overhang that could be used to generate the nucleosome core particle (NCP) to study the effect of repair enzymes on the lesions in the multicomponent cluster in more complex models.

## Conflicts of interest

The authors declare no conflicts of interest.

## Supplementary Material

RA-008-C8RA01992D-s001

## References

[cit1] Cooke M. S., Evans M. D., Dizdaroglu M., Lunec J. (2003). FASEB J..

[cit2] Gulston M., Fulford J., Jenner T., de Lara C., O'Neill P. (2002). Nucleic Acids Res..

[cit3] Juo S., Boorstein R. J., Teebor G. W. (1995). Nucleic Acids Res..

[cit4] Boiteux S., Guillet M. (2004). DNA Repair.

[cit5] Fortini P., Pascucci B., Parlanti E., D'Errico M., Simonelli V., Dogliotti E. (2003). Mutat. Res..

[cit6] Hegde M. L., Hazra T. K., Mitra S. (2008). Cell Res..

[cit7] Eot-Houllier G., Gonera M., Gasparutto D., Giustranti C., Sage E. (2007). Nucleic Acids Res..

[cit8] Jiang Y., Wang Y., Wang Y. (2009). Chem. Res. Toxicol..

[cit9] Blaisdell J. O., Wallace S. S. (2001). Proc. Natl. Acad. Sci. U. S. A..

[cit10] Shikazono N., Pearson C., O'Neill P., Thacker J. (2006). Nucleic Acids Res..

[cit11] Malyachuk S., Brame K. L., Youngblood R., Shi R., Harrison L. (2004). Nucleic Acids Res..

[cit12] Georgakilas A. G., O'Neill P., Stewart R. D. (2013). Radiat. Res..

[cit13] Cadet J., Davies K. A. (2017). Free Radicals Biol. Med..

[cit14] Krokan H. E., Nilsen H., Skorpen F., Otterlei M., Slupphaug G. (2000). FEBS Lett..

[cit15] David S. S., O'Shea V. L., Kundu S. (2007). Nature.

[cit16] Ho E. L., Parent M., Satoh M. S. (2007). J. Biol. Chem..

[cit17] Harrison L., Brame K. L., Geltz L. E., Landry A. M. (2006). DNA Repair.

[cit18] Eccles L. J., Lomax M. E., O'Neill P. (2010). Nucleic Acids Res..

[cit19] Kozmin S. G., Sedletska Y., Reynaud-Angelin A., Gasparutto D., Sage E. (2009). Nucleic Acids Res..

[cit20] Sage E., Shikazono N. (2017). Free Radicals Biol. Med..

[cit21] Eot-Houllier G., Eon-Marchais S., Gasparutto D., Sage E. (2005). Nucleic Acids Res..

[cit22] Dianov G. L., Thybo T., Dianova I. I., Lipinski L. J., Bohr V. A. (1999). J. Biol. Chem..

[cit23] Taylor J. S. (2015). DNA Repair.

[cit24] Esadze A., Rodriguez G., Cravens S. L., Stivers J. T. (2017). Biochemistry.

[cit25] Hill J. W., Hazra T. K., Izumi T., Mitra S. (2001). Nucleic Acids Res..

[cit26] Barone F., Dogliotti E., Cellai L., Giordano C., Bjřa M., Mazzei F. (2003). Nucleic Acids Res..

[cit27] Marenstein D. R., Chan M. K., Altamirano A., Basu A. K., Boorstein R. J., Cunningham R. P., Teebor G. W. (2003). J. Biol. Chem..

[cit28] Kypr J., Kejnovská I., Renčiuk D., Vorlíčková M. (2009). Nucleic Acids Res..

[cit29] Minetti C. A., Sun J. Y., Jacobs D. P., Kang I., Remeta D. P., Breslauer K. J. (2018). Biopolymers.

[cit30] Kung H. C., Bolton P. H. (1997). J. Biol. Chem..

[cit31] Zalesak J., Lourdin M., Krejci L., Constant J. F., Jourdan M. (2014). J. Mol. Biol..

[cit32] Crenshaw C. M., Wade J. E., Arthanari H., Frueh D., Lane B. F., Núñez M. E. (2011). Biochemistry.

[cit33] Breslauer K. J. (1994). Methods Mol. Biol..

[cit34] Minetti C. A., Remeta D. P., Iden C. R., Johnson F., Grollman A. P., Breslauer K. J. (2015). Biopolymers.

[cit35] Singh V., Kumari B., Das P. (2015). RSC Adv..

[cit36] Singh V., Kumari B., Maity B., Seth D., Das P. (2014). Mutat. Res..

