# Supplementary material for: Vicinal abasic site impaired processing of a Tg:G mismatch and 8-oxoguanine lesions in three-component bistranded clustered DNA damage
Source: RSC Adv. 2018 May 16;8(32):17921–6. doi: 10.1039/c8ra01992d (PMC9080475; doi:10.1039/c8ra01992d)
Supplement: RA-008-C8RA01992D-s001 [file RA-008-C8RA01992D-s001.pdf]

Electronic Supplementary Material (ESI) for RSC Advances.
This journal is © The Royal Society of Chemistry 2018

Vicinal abasic site Impair processing of Tg:G Mismatch and 8-Oxoguanine Lesions in Three-Component Bistranded Clustered DNA Damage

Bhavini Kumari[a], Pravin Jha[b], Kislay K.Sinha[b] and Prolay Das[a,*]

[a]Department of Chemistry, IIT Patna, Bihta, Patna-801103, Bihar, India

[b]Department of Biotechnology, NIPER Hajipur, Bihar, India

Supplementary Information

Material and Methods:

HPLC purified synthetic oligonucleotides possessing oxidative lesions as well as tetrahydrofuran (THF), d-spacer abasic site analog were obtained from Sigma custom oligo service. Uracil DNA glycosylase (UDG), hOGG1 and APE1 were purchased from New England Biolabs (NEB), Inc (USA). Recombinant hNTH1 enzyme was procured from Abcam (UK). Urea, formamide, glycerol, tris buffer and chemicals used for gel electrophoresis and buffer preparation were purchased from Sigma. SYBR Gold® was procured from Invitrogen. Polyacrylamide gel electrophoresis (PAGE) images were acquired with UVP Gel Doc-It 310 gel documentation system. The quantitative estimation of gel images was done with Vision Work Ls Image acquisition and analysis software from UVP (UK).

Hybridisation of oligonucleotides

Abasic site was generated by treating uracil containing double stranded oligonucleotide (0.1 nmol) with 5 units of UDG enzyme. Thus, Uracils serve as the site for abasic sites formation following UDG treatment. UDG enzyme treated samples were further confirmed for a complete generation of abasic site by incubation with 0.5 M NaOH for 30 min and visualized on a denaturing PAGE. The DNA duplexes with solitary as well as clustered oxidised lesions were constituted by annealing with its complementary strand in the presence of the 10 mM sodium phosphate buffer at pH 7.2.

Preparation of cell extract

The HeLa cell extract was prepared and protein concentration was determined by Bradford colorimetric method (6.0-6.5 mg/mL). The cell extracts were stored at −20 °C and used in further downstream experiments within a month without loss of activity.

Enzyme cleavage assay

The concentration of hOGG1 (50 ng) and hNTH1 (20 ng) enzyme used for incision assay of $\mathbf{B1\text{-}B2_{oG}}$ and $\mathbf{B1_{Tg}\text{-}B2}$, respectively for 1 picomol DNA was optimized by titration in previous study. Similar titration assay for 1 picomol of $\mathbf{B1\text{-}B2_{AP}}$ was done to optimize the amount of APE1 (0.15 ng) enzyme (Fig.S1).

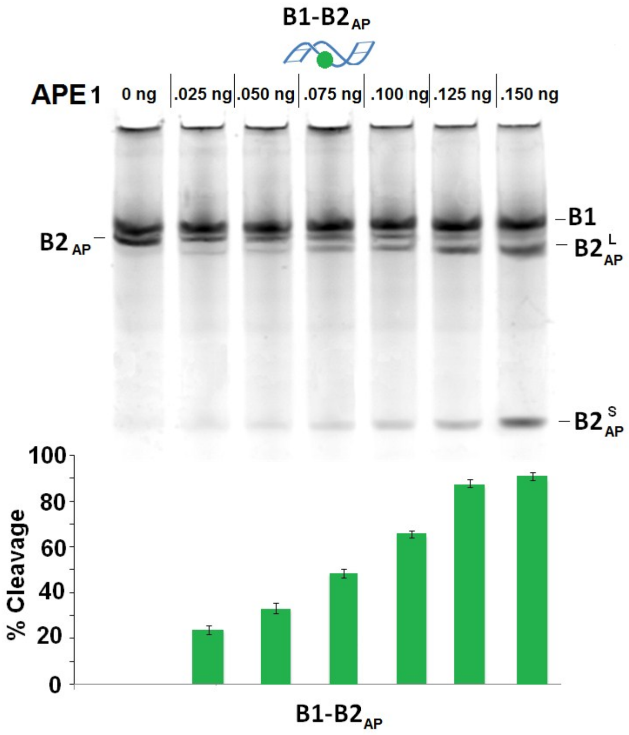

Fig. S1: 20% denaturing PAGE shows amount optimization for APE1 enzyme for cleavage of solitary abasic lesion in the **B1-B2$_{\mathbf{AP}}$** strand.

The hOGG1 enzyme (50 ng), hNTH1 enzyme (20 ng) and APE1 enzyme (0.15 ng) were mixed in proportion as optimized from enzyme titration for **B1$_{\mathbf{Tg}}$-B2$_{\mathbf{AP,oG}}$**. Enzyme incision assay with purified enzyme cocktail was conducted in the presence of mixture of hOGG1 enzyme (50 ng) and APE1 enzyme (0.15 ng) as well as mixture of hNTH1 enzyme (20 ng) and APE1 enzyme (0.15 ng) for 1 picomol of **B1$_{\mathbf{AP}}$-B2$_{\mathbf{oG}}$** and **B1$_{\mathbf{Tg}}$-B2$_{\mathbf{AP}}$**, respectively. Incision assays with cell extract were optimized (500 μg) for 1 pmol of **B1-B2$_{\mathbf{AP}}$** (Fig.S2).

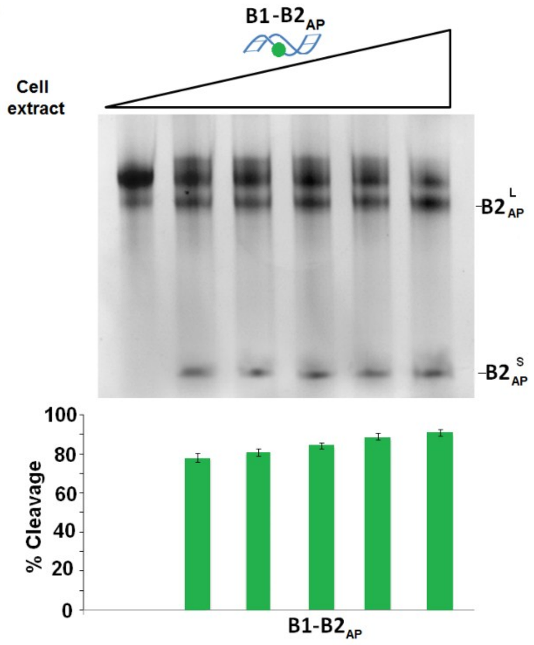

Fig. S2: 20% denaturing PAGE shows amount optimization of APE1 enzyme in cell extract for cleavage of solitary abasic site in **B1-B2$_{AP}$** strand.

The similar buffer composed of 10 mM $MgCl_2$, 1 mM DTT, 0.1 µg/µL bovine serum albumin, 20 mM Tris-HCl and 50 mM NaCl at pH 8 and 37 °C were used for all kind of titration studies in present study. The incubation time was 30 min and 60 min for assays carried out with cell extract and purified enzymes, respectively. Denaturation of all samples were done for 5 min at 90 ºC in denaturing stop solution (94% formamide, 0.1% bromophenol blue, 0.1% xylene cyanol and 10 mM EDTA pH 8) prior to load into the well of denaturing PAGE (20%, 29:1 acrylamide–bisacrylamide cross linking ratio, 7 M urea, 10% formamide at pH 8.0) for quantifying Single strand breaks (SSBs). Typically PAGE was run at 200 V for 2 h. The DSB formation was investigated in native PAGE with native loading buffer without heat shock step for non-denaturing sample.

Gel Staining and calculation of SSBs and DSBs

The quantitative evaluation of percentage cleavage at 8-oxodG, Tg and abasic site was done from Sybr gold® stained gel images.

Percentage of single strand breaks (SSBs) =

$$\frac{(Amount\ of\ fragment\ produced) \times 100}{(Amount\ of\ fragment\ produced + Amount\ of\ unreacted\ substrate)}$$

The following equation was used for the estimation of double strand breaks (DSBs) from native PAGE

Percentage of double strand breaks (DSBs) =

$$\frac{(Amount\ of\ Double\ strand\ break) \times 100}{(\ Amount\ of\ DSBs + Amount\ of\ unreacted\ substrate)}$$

Band shift assay:

The band shift assays were performed by incubating 1 pmol of the duplexes with 50 ng of hOGG1 in buffer at 37 °C for 30 min. Aliquots of the reaction were loaded on non-denaturing 20% PAGE in TTE buffer.

Repair assay:

Double stranded oligonucleotides (1 picomol) were incubated with 500 µg of cell extracts in repair buffer at 37 °C for 5, 10, 15, 20 and 30 min of time. The repair buffer was prepared from 0.1 mM each of dNTPs (dATP, dCTP, dGTP, dTTP), 10 mM $MgCl_2$, 10 mM DTT, 40 mM phosphocreatine and 1.6 µg/mL phosphocreatine kinase enzyme. The reaction were stopped after intended interval of time as mentioned above by adding non denaturing stop buffer (75% glycerol, 0.025% bromophenol blue, 0.025% xylene cyanol) and introduced into the wells of native PAGE (20%, 29:1 acrylamide–bisacrylamide cross linking ratio formamide pH 8.0) and run on native PAGE in TTE buffer.

Calculation for percentage of Restoration:

Titration assay with cell extract gave information about contribution of the relative amount of repair enzyme present in cell extract. Since, no radiolabelling was done, individual contributor of rejoined DNA fragment could not be established. The percentage restoration was calculated as the ratio of DSB remaining after 30 min incubation with cell extract containing repair buffer to the amount of DSB created by cell extract without repair buffer in 30 min in native PAGE. It is to be noted that restoration study was done by adding cell extract and repair buffer simultaneously for a maximum of 30 min.

In the case of the DSBs calculation during repair, yield of the DSBs is calculated as the ratio of total amount of the fragmented duplex to the sum of fragmented duplex and full length unreacted duplex.

Circular Dichroism (CD)

CD spectra of oligonucleotide were obtained on Jasco J-1500 in 10 mM sodium phosphate buffer (pH=7.0) in a total volume of 200 µL with 1 mm pathlength cuvette. The sample was scanned thrice with a scan rate of 50 nm/ min and data was articulated as degrees of ellipticity, in units of millidegrees (mdeg).

Thermal denaturation

Cecil 2025 UV-vis spectrophotometer was used to measure thermal melting using cuvette with 1.0 cm path length and 100 µl volume. 5 µM oligonucleotide were gradually heated from 20 °C to 90 °C at the rate of 0.5°C/min in buffer composed of 10 mM sodium phosphate, 1mM EDTA and 50 mM NaCl at pH 8.0 to find out thermal melting profile. The melting temperature (T_m) was obtained from first derivative of melting curves.

The thermodynamic parameters like enthalpy change (ΔH), entropy change (ΔS) and Gibbs' free energy change (ΔG) were obtained from curve fitting method which employed upon alpha curve and van't Hoff plots (ln K_T versus T^{-1}). The fraction (α_T) of oligonucleotide that remained hybridised in the duplex at a particular temperature T is implies as

$$\alpha_T = \frac{A_s - A}{A_s - A_d}$$

where, A_s and A_d is absorbance of oligonucleotide in fully denatured and totally hybridized condition respectively. A is absorbance at a specific point at temperature T on the thermal melting curve. The common expression for equilibrium constant equation where, C_{ts} signifies total concentration of strands where each individual strand present in equal concentration. Assuming a two-state model where n=2,

$$K_T = \frac{2\alpha_T}{(1-\alpha_T)^2 C_{ts}}$$

The van't Hoff plot ln K_T versus T^{-1} is straight line represented by

$$\ln K_T = \left[\frac{-\Delta H^\circ}{R}\right]\frac{1}{T} + \frac{\Delta S^\circ}{R}$$

Hence, value of ΔH^0 and ΔS^0 were derived from slope and Y-intercept of van't Hoff plot respectively. The value of universal gas constant (R) is 1.986 cal/mol K. ΔG^0 was calculated from the below given equation.

$$\Delta G^\circ = -RT \ln K_T = \Delta H^\circ - T\Delta S^\circ$$

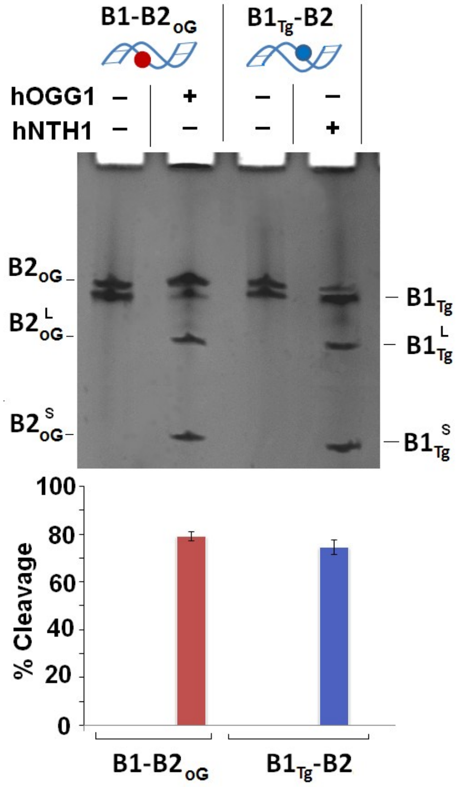

Fig. S3: 20% denaturing PAGE shows SSBs formation in **B1-B2$_{oG}$** and **B1$_{Tg}$-B2** oligonucleotides by lesion specific enzymes hOGG1 and hNTH1 , respectively.

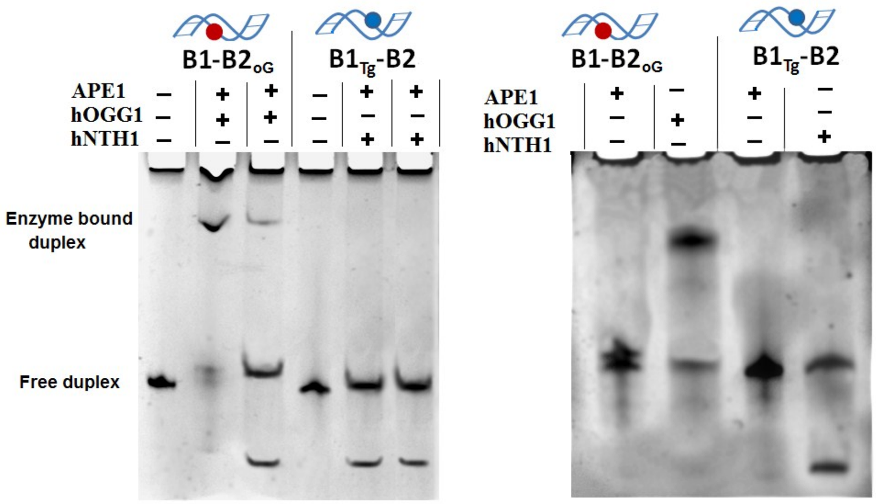

Fig. S4: 20% native PAGE presents a band shift assay to show the relative ease for approaching hOGG1 enzyme to 8-oxodG lesion in **B1-B2$_{oG}$**, **B1$_{AP}$-B2$_{oG}$** and **B1$_{Tg}$-B2$_{oG}$** oligonucleotides along with a specific binding of the **B1-B2** with hOGG1 enzyme.

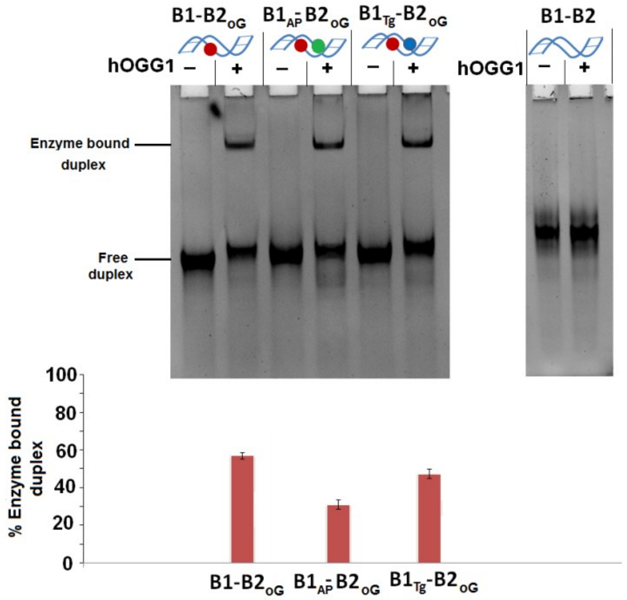

Fig. S5: 20% polyacrylamide gel shows enzyme bound complex in **B1-B2$_{oG}$** with the hOGG1 enzyme which diminishes after a sequential increase of APE1 enzyme. Such band shift has not observed in **B1$_{Tg}$-B2**.

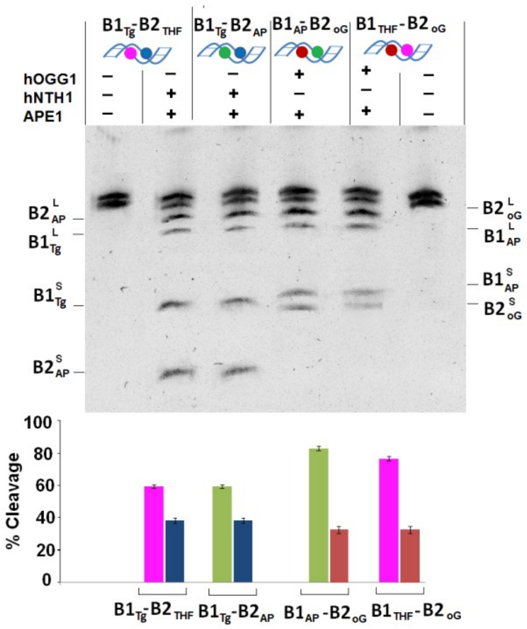

Fig. S6: 20% denaturing PAGE showing SSBs formation in **$B1_{Tg}$-$B2_{THF}$** & **$B1_{Tg}$-$B2_{AP}$** with cocktail of hNTH1 and APE1 and in **$B1_{THF}$-$B2_{oG}$** & **$B1_{AP}$-$B2_{oG}$** with cocktail of APE1 and hOGG1 with their respective control at 200 V. Three independent measurements (n=3) were used to calculate the standard deviation (SD). Each value in error bar represents mean ± SD.

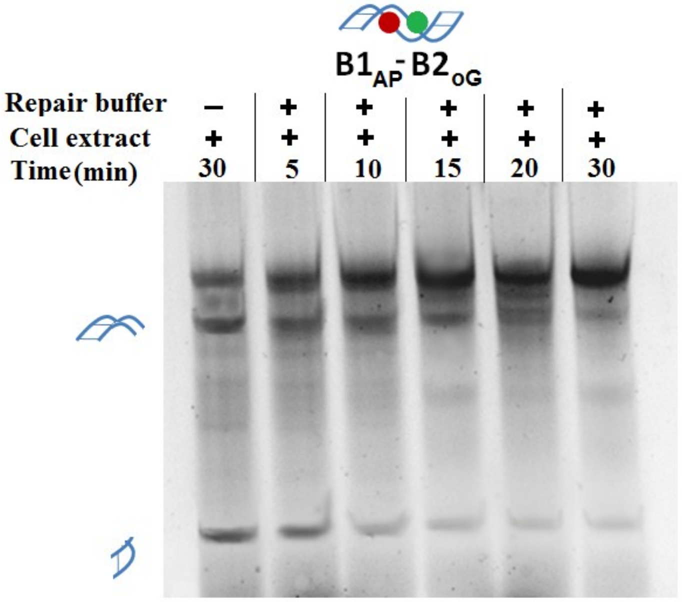

Fig. S7: 20% native PAGE shows the rejoining of cleaved sites in the case of **$B1_{AP}$-$B2_{oG}$**. The lane 1 shows the DSBs generated due to incubation of **$B1_{Tg}$-$B2_{oG}$** with cell extract for 30 min. The lane 6 shows the final DSBs remained in **$B1_{Tg}$-$B2_{oG}$** after incubation with cell extract and repair buffer for 30 min.

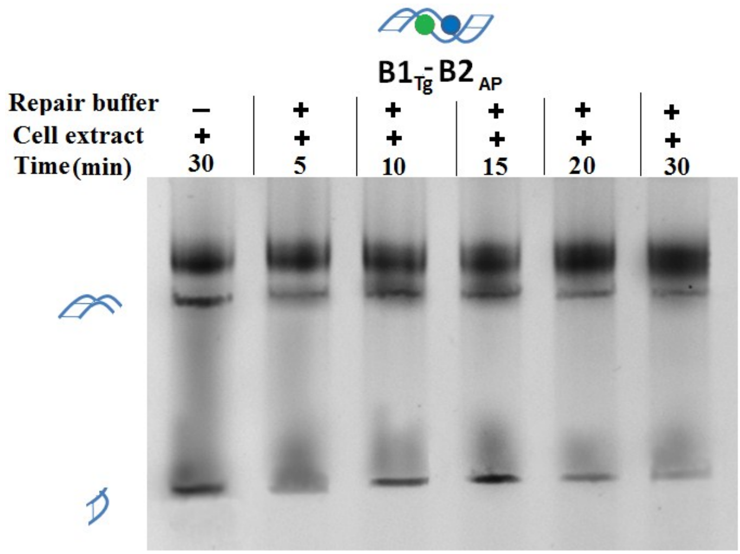

Fig. S8: 20% native PAGE shows the rejoining of cleaved sites in the case of **$B1_{Tg}$-$B2_{AP}$**. The lane 1 shows the DSBs generated due to incubation of **$B1_{Tg}$-$B2_{AP}$** with cell extract for 30 min. The lane 6 shows the final DSBs remained in **$B1_{Tg}$-$B2_{AP}$** after incubation with cell extract and repair buffer for 30 min.

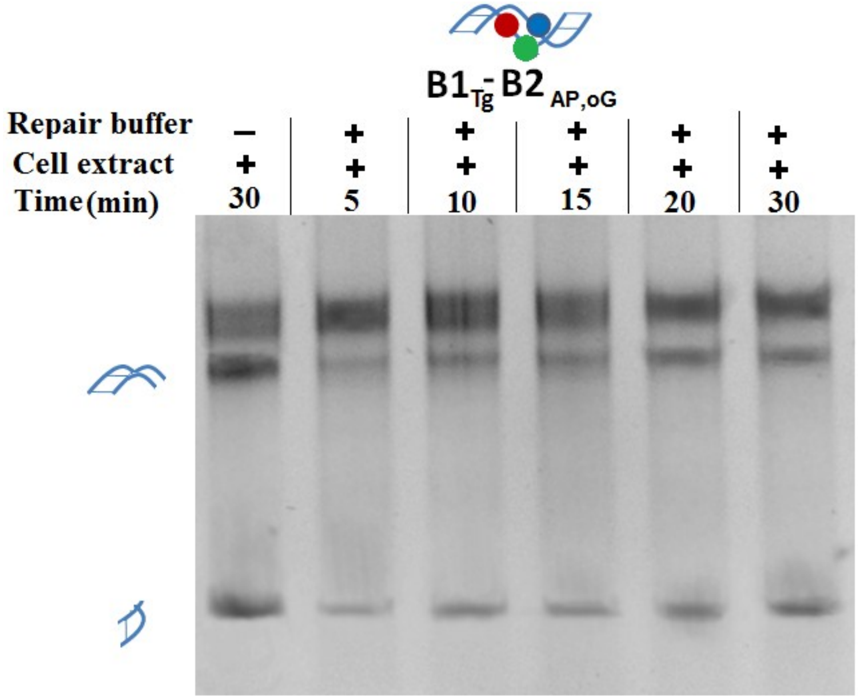

Fig. S9: 20% native PAGE shows the rejoining of cleaved sites in the case of **B1Tg-B2AP, oG**. The lane 1 shows the DSBs generated due to incubation of **B1Tg-B2AP, oG** with cell extract for 30 min. The lane 6 shows the final DSBs remained in **B1Tg-B2AP, oG** after incubation with cell extract and repair buffer for 30 min.

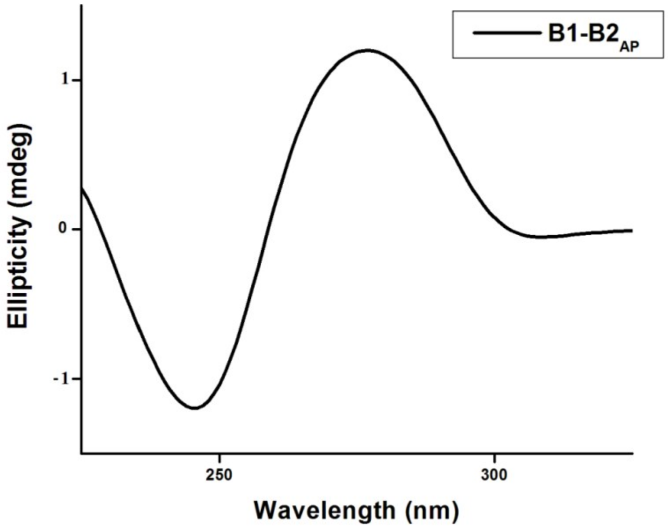

Fig. S10: Circular Dichroism spectra of **B1-B2$_{AP}$** strand embrace solitary abasic site and served as control.

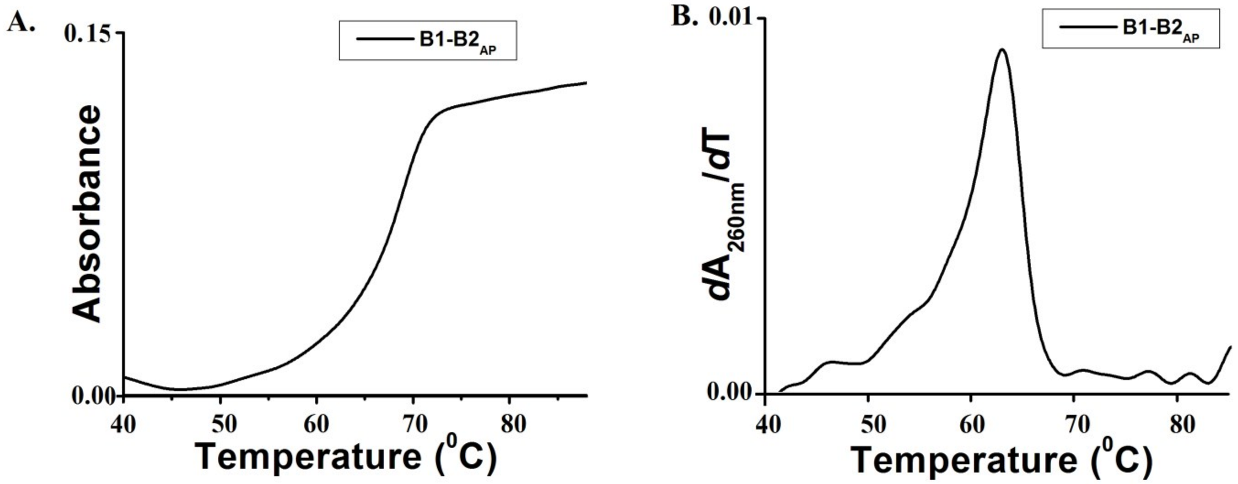

Fig. S11: (A) Thermal melting curve of **B1-B2$_{AP}$ (B)** First differential of thermal melting curve of **B1-B2$_{AP}$**

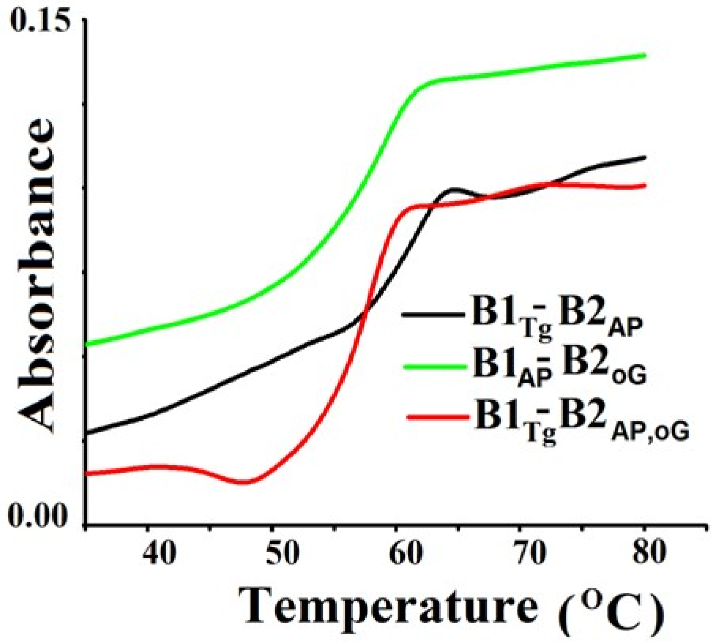

Fig. S12: Thermal melting curve of **$B1_{Tg}$-$B2_{AP}$**, **$B1_{AP}$-$B2_{oG}$** and **$B1_{Tg}$-$B2_{AP,oG}$**

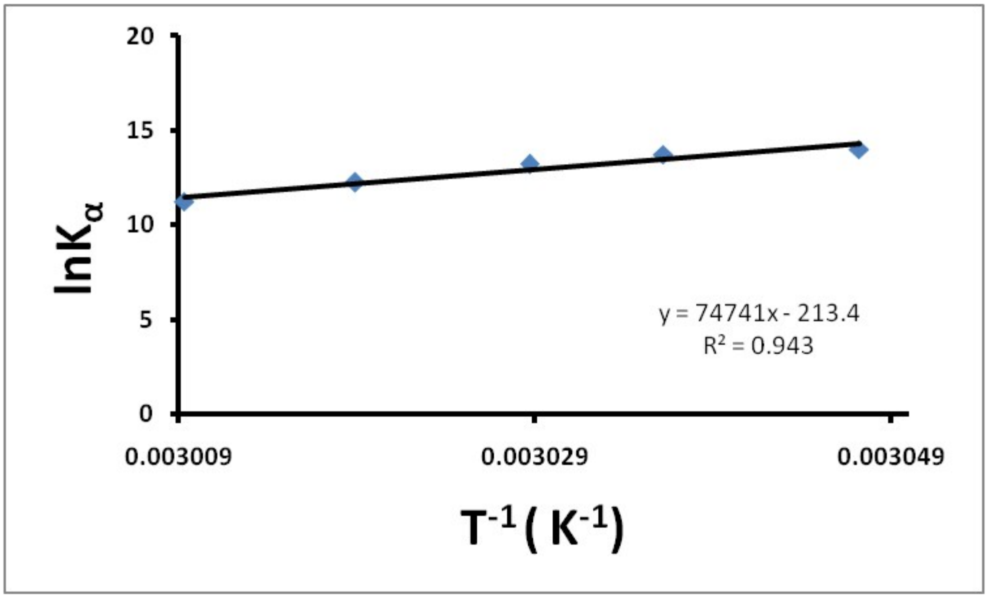

Fig. S13 The van't Hoff plot between $\ln K_{\alpha}$ and T^{-1} (K^{-1}) for **$B1$-$B2_{AP}$**. The slope of the graph gives the value of **ΔH^0** and intercept of the graph gives the value of **ΔS^0**.

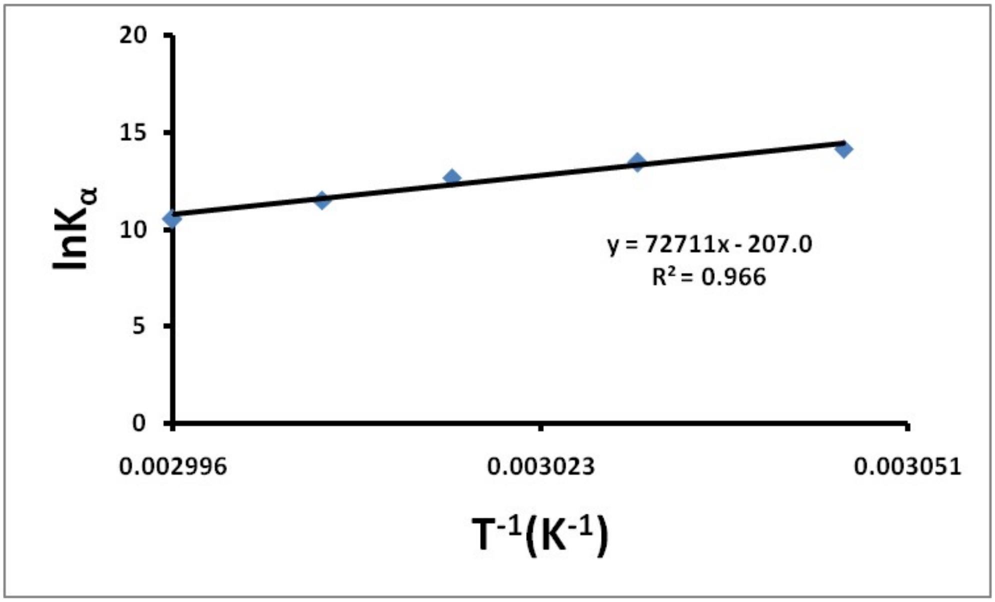

Fig. S14 The van't Hoff plot between lnK_α and T^{-1} (K^{-1}) for **B1$_{AP}$-B2$_{oG}$**. The slope of the graph gives the value of **ΔH^0** and intercept of the graph gives the value of **ΔS^0**.

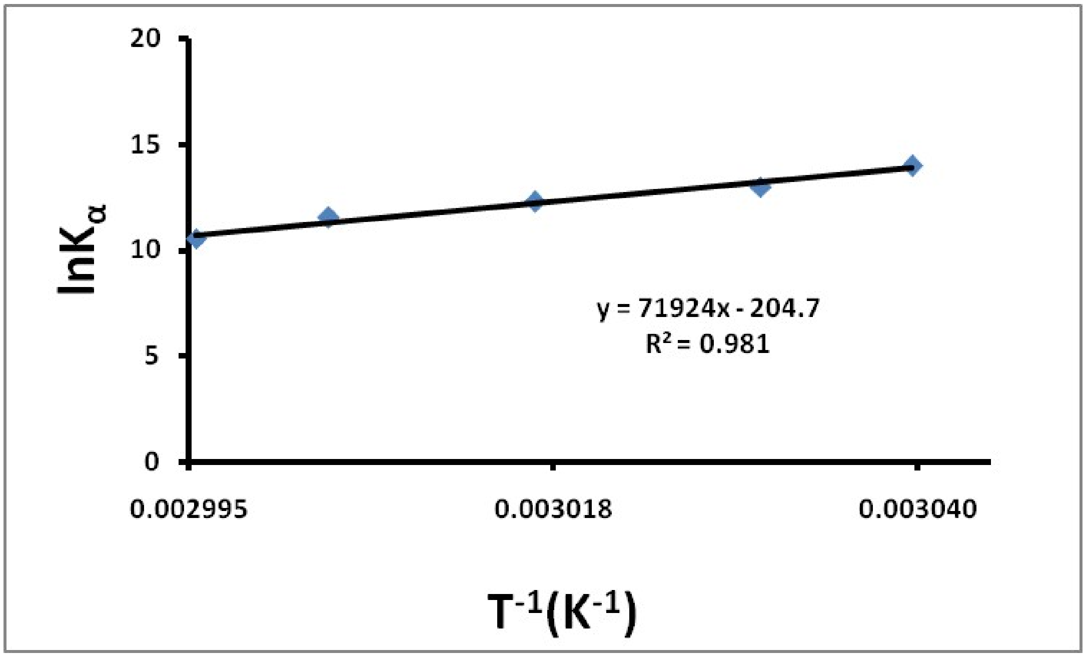

Fig. S15 The van't Hoff plot between lnK_α and T^{-1} (K^{-1}) for **B1$_{Tg}$-B2$_{AP}$**. The slope of the graph gives the value of **ΔH^0** and intercept of the graph gives the value of **ΔS^0**.

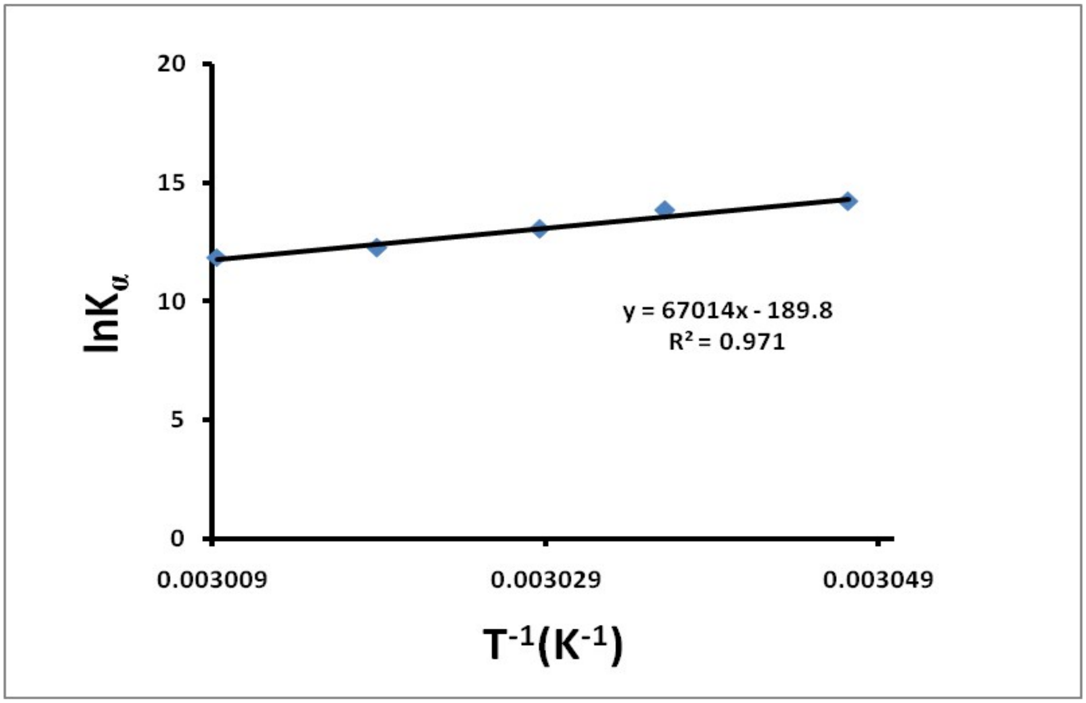

Fig. S16 The van't Hoff plot between lnK_{α} and T^{-1} (K^{-1}) for **B1$_{Tg}$-B2$_{AP,oG}$**. The slope of the graph gives the value of **ΔH^0** and intercept of the graph gives the value of **ΔS^0**.
